# Umami synergy as the scientific principle behind taste-pairing champagne and oysters

**DOI:** 10.1038/s41598-020-77107-w

**Published:** 2020-11-18

**Authors:** Charlotte Vinther Schmidt, Karsten Olsen, Ole G. Mouritsen

**Affiliations:** Department of Food Science, Taste for Life and Design and Consumer Behavior, University of Copenhagen, Rolighedsvej 26, 1958 Frederiksberg, Denmark

**Keywords:** Biophysics, Chemistry

## Abstract

Food and flavour pairing are commonly used as an empirically based phenomenology by chefs and food innovators for creating delicious dishes. However, there is little if any science behind the pairing systems used, and it appears that pairing is determined by food culture and tradition rather than by chemical food composition. In contrast, the pairing implied by the synergy in the umami taste, elicited by free glutamate and free nucleotides, is scientifically founded on an allosteric action at the umami receptor, rendering eggs-bacon and cheese-ham delicious companions. Based on measurement of umami compounds in champagnes and oysters we suggest that a reason why champagne and oysters are considered good companions may be the presence of free glutamate in champagne, and free glutamate and 5′-nucleotides in oysters. By calculations of the effective umami potential we reveal which combinations of oysters and champagnes lead to the strongest umami taste. We also show that glutamate levels and total amount of free amino acids are higher in aged champagnes with long yeast contact, and that the European oyster (*Ostrea edulis*) has higher free glutamate and nucleotide content than the Pacific oyster (*Crassostrea gigas*) and is thus a better candidate to elicit synergistic umami taste.

## Introduction

Humans’ food preferences are complex and dependent on a wide range of factors, such as food culture, tradition, social upbringing, as well as genetic and physiological differences^1–4^. The preferences for certain aromas, tastes, and textures behind food preferences are equally complex, although it is believed that we carry with us our distant ancestors’ quest for certain aroma compounds that during evolution has led us to nutritious and calorie-rich ripe fruit^5^. In addition, there are some fundamental and universal preferences across different individuals, populations, and human races, manifested in our common craving for the basic tastes sweet and umami, a drive that has formed us as omnivores during evolution to guide us to calorie- and protein-rich foods^6, 7^.

A further step in complexity arises when considering the way we combine food ingredients and their different sensory cues in dishes and meals: this is what cooking and the culinary arts are all about. In some cases, it is commonly known and accepted that certain food items are good companions, such as eggs and bacon or stilton and rhubarb, as well as certain food-beverage items, such as wine and cheese, the latter being a well-known pairing example that has been extensively investigated by sensory evaluation^8–10^. In other cases, inventive chefs have designed new combinations that have not been considered before, such as caviar and white chocolate^11^. This has led to the so-called Food Pairing Theory that claims to be able to explain good pairing as determined by an overlap of identical or related aroma components in the paired food items. The pairing principle has also been used for substitution of one food item by another, e.g., tomatoes by strawberries. It should be pointed out that there is no sound principle behind this Food Pairing Theory.

In a landmark work by Ahn et al.^12^, flavour network analysis using data-driven methods^13^ was applied to an extensive database of cuisine metadata (56,000 recipes) from around the world coupled to a database for volatile aroma compounds (1021 compounds). This led to the startling result that food pairing, based on joint aroma compounds (i.e., Food Pairing Theory), may only holds true in some cultures and not in others. Another study based on metadata pertaining to local Chinese cuisines found that geographical distance rather than climate similarity implied resemblance in the favoured local recipes^14^. In addition, other points of criticism have been raised against in the Food Pairing Theory. According to De Klepper^15^, first of all, it is a fact that humans do not perceive aroma compounds in the same manner as gas chromatographs. Specifically, output from gas chromatographs used in the Food Pairing Theory does not account for possible interrelating effects and threshold values of aroma compounds, which will be perceived by humans. Second, the theory tends to neglect the body of scientific literature that shows that food preferences and successful flavour combinations are for the most part learned or culturally determined. Third, there are very few studies testing whether two food items with overlapping aromatic compounds taste better than two foods that have fewer or no overlapping aromatic compounds. Hence it appears that the Food Paring Theory is flawed and not supported by available data.

Although there is no established scientific theory for food-paring pairing in general, a very large number of phenomenological systems and empirical principles have been suggested by chefs and sommeliers and described in the culinary literature. In a recent paper, Eschevins et al.^16^ reviewed the literature and reported results from interviews of French sommeliers and beer experts demonstrating that these professionals use pairing principles based on perceptual, conceptual, and affective categories when matching food with beverages. Overall, matching food with either wine or beer seems to rely on the same principles, but the adopted terminologies are not standardized, and the preferred matches appears to be based on norms, conceptual association, and social context.

Apart from the Food Pairing Theory, a number of studies have investigated food pairing by its chemical composition, some of which is highlighted in Lahne^17^. Tamura et al.^18^ demonstrated that fishy aftertaste in wine consumed with seafood was likely to be caused by the ferrous iron concentration in the wine, rendering white wines which are generally lower iron, a better match than red wines. A similar study by Fujita et al.^19^ showed that fishy off-flavours were caused by sulphur-dioxide present in wine, but not in sake, when consumed with seafood thus ascribing a better pairing with sake. In contrast to the flawed Food Pairing Theory and the phenomenological food-paring principles described above, there is a scientifically based and well-understood principle of *taste pairing* by chemical composition in form of synergetic effect of umami-taste compounds.

Umami taste is elicited by an allosteric action on the T1R1/T1R3 umami receptor that is a G-protein-coupled hetero-dimer protein located in the membranes of the taste cells^20^. The synergy is stimulated by the simultaneous binding of a free ion of amino acid glutamate (basal umami) and a free ion from certain 5′-ribonucleotides (synergetic umami) on the Venus-flytrap motif of the receptor^21^. This leads to a stronger binding of the glutamate ion and a super additive enhancement of the neural signal to the taste centre in the brain^22^ thus increasing the perceived intensity of umami taste when both free glutamate and nucleotides are present in a meal. Apart from yielding a desirable savoury taste, umami compounds can also affect the perception of other taste compounds, such as enhancing sweet taste and masking bitter, making it a desirable taste attribute in foods and beverages^7^.

Phenomenologically, this powerful enhancement of the umami sensation has been known in the Japanese cuisine for centuries and was scientifically first established as a principle by Kuninaka^23^ who discovered that a free ribonucleotide, guanylate (guanosine-5′-monophosphate, GMP), derived from the nucleic acid guanylic acid, enhances the sensation of free glutamate. This principle also explained the strong umami in the Japanese soup stock dashi prepared by combining aqueous extracts of a brown seaweed (konbu, *Saccharina japonica*) and a highly processed fish product (katsuobushi) that contain, respectively, free glutamate and another free nucleotide, inosinate (inosine-5′-monophosphate, IMP), derived from the nucleic acid inosinic acid. Free guanylate is present in dry shiitake mushrooms which traditionally have been used along with konbu for a vegan version of dashi^7^. It is also known that other free nucleotides can impart this synergistic effect, such as adenylate (adenosine-5′-monophosphate, AMP) and xanthosinate (xanthosine-5′-monophosphate, XMP). The relative umami taste activity of the different free nucleotides has been characterized by Yamaguchi et al.^24^.

Extracts of seaweed, fish, and fungi being a trait of the Asian cuisine, may for some seem strange ingredients for delicious food. However, the exact same umami-based pairing principle (glutamate-nucleotide) lies behind good companions in the Western cuisine, most notably meat-vegetables, eggs-bacon, cheese-ham, and tomato-beef^21, 25^. The synergistic effect of umami taste compounds may therefore be a universal principle, contributing to good food-pairing across culture, tradition, and geographical distance.

In the present paper we have investigated one of the most celebrated and classical food-beverage pairings in gastronomy: champagne and oysters. A longstanding question has been whether this kind of pairing is more art than science. Obviously, chefs and sommeliers know their trade and can rationalize the way they match food and beverages by contrast and affinity, matching oyster properties like meatiness, salinity, and fattiness with champagne properties like minerality, brininess, and prickling acidity. The concepts of nuttiness, savouriness, and umami have also been evoked, in particular in relating possible umami in beverages like champagne and some sherry wines with long yeast contact^26, 27^. Here we hypothesize that champagne and oysters are good companions in terms of taste because of their pairing may facilitate umami-synergy. We investigate our hypothesis by quantitative measurement of umami compounds in a great variety of champagnes and two different species of oysters. Good pairing of champagne and oysters is therefore hypothesized to be based on a sound scientific principle of synergistic umami effect. Obviously, other components such as aroma and texture are also important which we will return to in the discussion. But first, we present our data from an analysis of a series of champagnes with long yeast contact along with data for two types of Nordic oysters, European flat oyster (*Ostrea edulis*) and Pacific oyster (*Crassostrea gigas*).

## Results

### Free amino acids in champagnes

In Table 1 we present the free amino acid (FAA) profiles of a series of beverages, in particular genuine French champagnes, along with a reference sample of sparkling wine with presumed short yeast contact for comparison. Asp and Glu are the primary amino acids responsible for umami taste, but also other FAA, such as Gly, Ala, Ser, Thre and Pro, are important to taste, since these amino acids are known to elicit sweet taste, while other elicit bitter taste^28, 29^.

**Table 1:** Mean values ($\stackrel{-}{\mathrm{x}}$) of free amino acids (n = 17) in champagnes (n = 17) and sparkling wine (n = 1) in mg/100 mL based on a sample triplicate (n = 3) ± standard deviation (σ).

Champagne	Ref		Umami		Sweet					Bitter									Neutral	
			Glu	Asp	Pro	Gly	Ala	Thr	Ser	His	Arg	Val	Met	Trp	Phe	Ile	Leu	Lys	Tyr	Sum
*AR Lenoble*. Chonilly. Blancs de blancs. MAG 14, Grand cru. Non-vintage	C01	$\stackrel{-}{\mathrm{x}}$	7.5	4.7	46.5	2.7	15.2	2.7	3.5	0.7	4.8	1.9	0.7	n.d	2.9	1.0	3.6	5.3	1.8	106
		σ	± 0.1	± 0.1	± 2.5	± 0.1	± 0.3	± 0.1	± 0.1	± 0.1	± 0.1	± 0.0	± 0.0	–	± 0.1	± 0.0	± 0.1	± 0.1	± 0.0	
*Duval-Leroy 2000*. Femme de Champagne. Vintage 2000	C02	$\stackrel{-}{\mathrm{x}}$	7.3	3.2	30.0	4.0	18.4	n.d	3.2	0.6	5.8	n.d	6.5	n.d	3.8	0.3	5.0	8.7	n.d	97
		σ	± 0.2	± 2.6	± 1.9	± 0.1	± 0.4	–	± 0.1	± 0.0	± 0.2	–	± 0.6	–	± 0.4	± 0.2	± 0.2	± 0.0	–	
*Guy Charlemagne*. Blanc de Blancs. Grand Cru. Extra brut. Vintage 2008	C03	$\stackrel{-}{\mathrm{x}}$	7.0	6.1	30.0	3.0	16.3	n.d	3.9	0.6	13.4	0.0	1.9	n.d	1.9	0.0	3.7	5.9	1.5	95
		σ	± 0.1	± 0.3	± 7.5	± 0.2	± 0.8	–	± 2.3	± 0.2	± 0.5	± –	± 1.2	–	± 0.4	–	± 0.4	± 0.7	± 0.2	
*Tattinger*. Millésimé Récolte 2000. Brut. Vintage 2000	C04	$\stackrel{-}{\mathrm{x}}$	7.0	10.6	26.9	3.7	12.8	n.d	3.5	1.2	14.8	2.1	2.2	n.d	3.7	0.5	5.6	9.1	1.8	106
		σ	± 0.1	± 0.8	± 2.8	± 0.1	± 0.2	–	± 0.1	± 0.7	± 0.2	± 0.0	± 0.2	–	± 0.1	± 0.0	± 0.0	± 0.1	± 0.0	
*Duval-Leroy*. Femme. Grand Cru. Brut. Non-vintage	C05	$\stackrel{-}{\mathrm{x}}$	5.6	1.9	33.9	2.9	8.8	n.d	1.6	n.d	2.7	n.d	5.7	n.d	1.1	n.d	3.3	6.2	n.d	74
		σ	± 0.4	± 0.2	± 2.8	± 1.7	± 0.7	–	± 0.2	–	± 0.2	–	± 0.4	–	± 0.2	–	± 0.3	± 0.5	–	
*Duval-Leroy*. Rosé Prestige. Brut. Non-Vintage	C06	$\stackrel{-}{\mathrm{x}}$	4.3	3.1	50.7	2.2	8.2	0.8	1.3	0.6	4.5	1.5	0.3	n.d	1.9	0.6	2.8	4.3	1.0	88
		σ	± 0.3	± 0.6	± 14.1	± 0.1	± 0.2	± 0.0	± 0.0	± 0.0	± 0.4	± 0.0	± 0.1	–	± 0.4	± 0.1	± 0.1	± 0.1	± 0.6	
*Clement Perseval*. Rosé. Non-vintage. Extra brut. France	C07	$\stackrel{-}{\mathrm{x}}$	4.3	2.2	24.0	1.5	6.9	0.3	2.2	n.d	2.9	0.4	n.d	n.d	1.1	n.d	2.3	3.8	0.5	52
		σ	± 1.8	± 0.9	± 14.1	± 0.2	± 0.3	± 0.2	± 0.2	–	± 2.6	± 0.1	–	–	± 0.0	–	± 0.0	± 0.1	± 0.3	
*Jacquesson 738*. Extra brut. Non-vintage	C08	$\stackrel{-}{\mathrm{x}}$	4.0	3.0	26.6	2.1	6.5	n.d	1.4	0.5	5.1	n.d	2.7	n.d	1.1	n.d	2.7	4.5	n.d	60
		σ	± 0.3	± 0.4	± 2.7	± 0.3	± 3.8	–	± 0.3	± 0.3	± 0.8	–	± 2.2	–	± 0.4	–	± 0.5	± 0.4	–	
*Legras & Haas*. Chonilly. Blancs de blancs. Vintage 2011	C09	$\stackrel{-}{\mathrm{x}}$	3.9	5.6	31.8	1.9	4.4	1.0	1.5	1.3	2.0	1.6	0.5	n.d	2.8	1.0	4.0	5.6	2.1	71
		σ	± 0.1	± 0.1	± 0.8	± 0.1	± 0.1	± 0.0	± 0.0	± 0.0	± 0.0	± 0.0	± 0.0	-	± 0.0	± 0.0	± 0.0	± 0.1	± 0.0	
*Duval-Leroy*. Brut. Non-vintage	C10	$\stackrel{-}{\mathrm{x}}$	3.8	2.6	33.9	2.9	10.4	n.d	1.8	n.d	1.5	n.d	5.1	n.d	1.4	n.d	3.1	6.0	n.d	72
		σ	± 0.4	± 0.3	± 4.0	± 0.2	± 0.9	–	± 0.3	–	± 0.1	–	± 0.5	–	± 0.9	–	± 0.3	± 0.6	–	
*Lanson*. Black Label. Brut. Non-vintage	C11	$\stackrel{-}{\mathrm{x}}$	3.7	3.4	20.7	2.3	7.5	n.d	1.6	0.6	14.5	0.4	n.d	n.d	1.0	n.d	2.2	4.4	n.d	62
		σ	± 0.2	± 0.3	± 0.4	± 0.1	± 0.4	–	± 0.1	± 0.4	± 0.6	± 0.2	–	–	± 0.1	–	± 0.1	± 0.1	–	
*Duval-Leroy*. Fleur de Champagne. Premier cru. Brut. Non-vintage	C12	$\stackrel{-}{\mathrm{x}}$	3.7	2.7	26.3	2.4	9.8	n.d	1.5	n.d	4.4	n.d	7.0	n.d	1.7	n.d	2.3	4.6	n.d	67
		σ	± 0.2	± 1.6	± 1.1	± 0.1	± 0.3	–	± 0.1	–	± 0.1	–	± 0.4	–	± 0.3	–	± 0.0	± 0.1	–	
*Paul Déthune*. Brut. Non-vintage	C13	$\stackrel{-}{\mathrm{x}}$	2.4	1.8	16.3	1.4	3.2	n.d	0.7	n.d	6.8	n.d	2.6	n.d	0.7	n.d	1.8	3.4	n.d	41
		σ	± 0.1	± 0.1	± 0.2	± 0.1	± 0.2	–	± 0.0	–	± 0.3	–	± 1.5	–	± 0.4	–	± 0.3	± 0.1	–	
*Déthune*. Grand Cru. Brut. Vintage 2008	C14	$\stackrel{-}{\mathrm{x}}$	2.2	3.8	18.6	1.8	3.7	n.d	1.0	n.d	4.6	n.d	3.5	n.d	1.1	n.d	2.5	4.1	0.6	48
		σ	± 0.2	± 0.5	± 2.3	± 0.2	± 0.4	–	± 0.1	–	± 0.5	–	± 2.0	–	± 0.3	–	± 0.4	± 0.5	± 0.3	
*Savart*. Accomplie. Extra brut. Non-vintage	C15	$\stackrel{-}{\mathrm{x}}$	1.8	1.6	18.2	1.5	2.6	n.d	0.6	n.d	4.1	n.d	1.0	n.d	0.7	n.d	1.2	3.5	n.d	37
		σ	± 0.3	± 0.3	± 1.3	± 0.1	± 0.3	–	± 0.1	–	± 0.4	–	± 0.6	–	± 0.4	–	± 0.2	± 0.4	–	
*Pascal Mazet*. Chigny les Roses. Brut. Vintage 2006	C16	$\stackrel{-}{\mathrm{x}}$	1.7	1.4	16.9	1.5	2.9	n.d	0.6	n.d	0.7	n.d	1.9	n.d	0.5	n.d	1.8	3.4	n.d	33
		σ	± 0.1	± 0.2	± 0.8	± 0.0	± 0.0	–	± 0.4	–	± 0.1	–	± 0.8	–	± 0.3	–	± 0.2	± 0.1	–	
*Duval-Leroy*. Demi-sec. Non-vintage	C17	$\stackrel{-}{\mathrm{x}}$	1.4	2.0	29.0	2.0	5.4	n.d	1.0	n.d	6.1	n.d	3.6	n.d	2.1	n.d	2.6	5.2	n.d	60
		σ	± 0.0	± 0.1	± 1.1	± 0.0	± 0.1	–	± 0.1	–	± 0.2	–	± 0.6	–	± 1.3	–	± 0.1	± 0.1	–	
*Savart*. Xpression. Nature. Vintage 2012	C18	$\stackrel{-}{\mathrm{x}}$	n.d	n.d	15.0	1.1	0.9	n.d	n.d	n.d	1.4	n.d	1.2	n.d	n.d	n.d	n.d	1.7	n.d	21
		σ	–	–	± 0.8	± 0.1	± 0.0	–	–	–	± 0.1	–	± 0.5	–	–	–	–	± 0.0	–	
Verdi (reference sample)	W01	$\stackrel{-}{\mathrm{x}}$	n.d	n.d	27.8	n.d	1.0	1.6	n.d	n.d	1.4	n.d	4.7	n.d	n.d	n.d	n.d	1.5	n.d	38
		σ	–	–	± 2.4	–	± 0.1	± 0.1	–	–	± 0.1	–	± 0.7	–	–	–	–	± 0.1	–	

The samples are listed according to descending concentration of glutamic acid (Glu). All listed beverages are genuine French champagnes, unless other county of origin is stated. *Sum* denotes sum of mean of all FAA. *n.d.* = not detected, i.e. < limit of detection (LoD) ≤ 1 μM.

The free amino acid analysis identifies Glu in all samples of champagne, except for C18, whereas it for the reference sample, a sparkling wine (Verdi, Italy) is not identified. The largest contents of Glu are found in *AR Lenoble*, and in some of the older champagnes, *Guy Charlemange*, vintage 2008, *Duval-Leroy*, vintage 2000, and *Tattinger*, vintage 2000 (cf. C01–C4, Table 1). From the analysed concentrations of Glu, it is found that the concentrations are below the taste threshold limit of basal umami taste, 29–30 mg/100 mL^30^ in all beverage samples, meaning that these beverages may not elicit umami taste by themselves, discounting the possible presence of other potential umami taste compounds not characterised in the present study. In addition to the content of Glu, the sum of FAA (n = 17, Asp, Glu, Ser, Gly, Thr, Ala, Pro, His, Arg, Val, Met, Trp, Phe, Ile, Leu, Lys, and Tyr) is shown for all samples (Table 1). The average sum of FAA in the analysed champagnes (reference sample excluded) is 65 mg/100 mL. Most of the samples of champagne have a significantly higher sum of FAA compared to the concentration of 38 mg/100 mL found in the sparkling wine reference sample, except for C18, C16, and C15, which have a lower amount of FAA than the sparkling wine sample. It is further noticed that the highest-ranking sample in terms of Glu content also contains the largest sum of FAA. Besides it can be seen, that a relatively high amount of sweet tasting amino acids Pro and Ala was characteristic for most of the champagne samples (Table 1).

**Figure 1:**
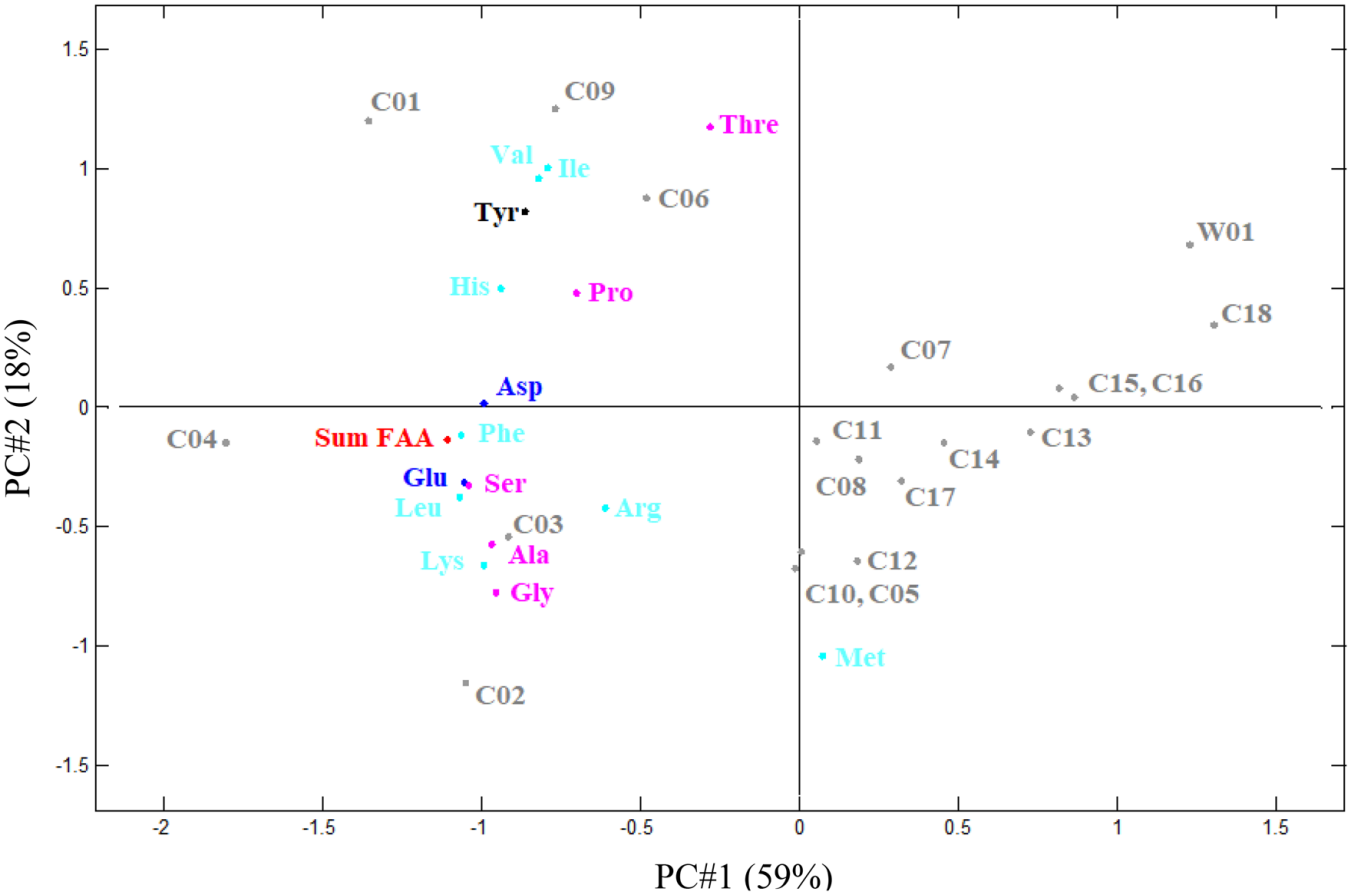
PCA bi-plot of scores and loadings plot, PC1 vs. PC2, describing 59% and 18% of the variance between samples, respectively. C01-C18 denote the champagnes listed in Table 1, numbered after descending content of Glu and W01 denotes the sparkling wine (reference sample). The scores (FAA) are coloured according to basic taste properties: blue = umami taste, pink = sweet taste, turquoise = bitter, black = neutral, and red = sum of FAA.

The FAA data from Table 1 is presented in a Principal Component Analysis (PCA) bi-plot with scores and loadings in Fig. 1, displaying the variance between the samples. The first principal component (PC1) accounts for 59% of the variance between samples, and the second principal component (PC2) accounts for 18%. It is noticed that certain samples are more similar than others on account of their FAA contents, where C02–04 are dominated by umami and sweet tasting amino acids, and C01, C06, and C09 are dominated by sweet and bitter tasting FAA (Fig. 1).

The remaining samples of champagnes are more or less similar to the reference sample of sparkling wine, the closest ones being C15, C16 and C18, which are together with the sparkling wine reference positioned in the PCA bi-plot (Fig. 1) to be least dominated by umami tasting amino acids, Asp and Glu, as well as sweet tasting Ser, Ala and Gly, also evident from the data presented in Table 1. Other mid- and low-ranking champagnes in terms of Glu content are found to be the less dominated by FAA in general and thereby the sum of FAA, rather than specific FAA taste properties (umami, sweet, bitter), indicating that a high content of overall FAA may suggest enhanced taste properties.

### Free amino acids and nucleotides in oysters

In Table 2 we present the results for the contents of selected FAA (Glu, Asp, Ser, Gly, Thr, Ala and Pro) in two species of oysters, the flat European oyster (*Ostrea edulis*) and the Pacific oyster (*Crassostrea gigas*), harvested in the same waters at a location in Denmark.

**Table 2:** Free amino acids (n = 7) in oysters, eliciting umami taste (Asp, Glu) and sweet taste (Ser, Gly, Thr, Ala, Pro) and Sum of FAA (n = 17). Values listed are in mg/100 g or mg/100 mL ± standard deviation.

Oyster	Free amino acids								Water	
	Glu	Asp	Pro	Gly	Ala	Thr	Ser	Sum*	%	mL
**Solid**										
European oyster (*Ostrea edulis*)	256.6	206.1	31.3	70.1	2830.1	n.d	16.8	4229.0	75.5	
	± 13.3	± 18.7	± 25.8	± 70.8	± 41.3	–	–			
Pacific oyster (*Crassostrea gigas*)	160.1	55.3	65.5	157.4	2506.6	49.6	13.8	3444.8	80.4	
	± 2.3	± 9.0	± 17.3	± 1.0	± 32.5	± 2.0	± 3.2			
**Liquid**										
European oyster (*Ostrea edulis*)	14.4	15.4	n.d	12.4	167.9	2.2	0.7	259.2		12.0
	± 0.5	± 1.3	–	± 0.3	± 3.7	± 0.1	± 0.1			
Pacific oyster (*Crassostrea gigas*)	2.2	2.3	n.d	5.0	50.6	0.7	n.d	73.1		58.5
	± 0.2	± 0.7	–	± 0.3	± 2.5	± 0.1	–			

The water content in percent (%) is given for solid part (whole body) and water content in mL is given for the liquid part (free floating water) per 3 oysters. Sum denotes sum of mean of all FAA (n = 17). *n.d.* = not detected, i.e. < limit of detection (LoD) ≤ 1 μM.

The levels of FAA are found to be much higher in oysters than in the analysed samples of champagnes in Table 1. The major finding from Table 2 is that when it comes to the content of umami tasting FAA, i.e., Glu and Asp, in both the free flowing water part (liquid) and in the whole body part (solid) of *Ostrea edulis,* it is found to be considerably higher than in the corresponding parts of *Crassostrea gigas.* Specifically, *Ostrea edulis* contains about 60% more free Glu in the solid part than *Crassostrea gigas.* It is noted for both types of oysters that the solid part contains more of a given free amino acid than the liquid part. It is also noteworthy, that the water content in the solid part of *Crassostrea gigas* is higher than in *Ostrea edulis* and that the volume of the liquid part is almost five times larger (Table 2). Ostreophiles know that the taste experience of oysters involves many other aspects than the actual taste, not least the tactile sensation and mouthfeel of the meaty part of the oyster. Pacific oysters are often ascribed a robust, full-bodied flavour whereas European oysters are considered milder full-bodied with a slight metallic finish^31^. Some connoisseurs describe the texture of the European oysters as more firm and less squishy which may be partly ascribed to the lower water content and the lower amount of liquid.

Table 3 presents the analysed contents of free nucleotides (IMP, GMP, XMP, AMP, and UMP) in the two types of oysters. UMP and AMP are found in the highest concentrations, GMP and IMP are present in low concentrations, while XMP was below detection threshold. To the extent of the present authors’ knowledge, UMP has not been reported to elicit umami taste and the umami taste contribution in oysters may therefore be considered to originate from GMP, IMP and AMP. The content of IMP and GMP is above recorded taste threshold values from Maga^32^ of 0.1 mg/100 g and 0.03 mg/100 g, respectively (no threshold values for AMP are available). The analysed contents of IMP and GMP from both types of oysters are thus capable of yielding synergistic umami taste with both its own content of free glutamate as well as the contents found in champagne.

**Table 3:** Free nucleotides (n = 5) in oysters in units of mg/100 g in the solid part (whole body) and in units of mg/100 mL in the liquid part (free flowing water part) ± standard deviation (σ) based on a sample triplicate.

Oyster	Nucleotides				
	IMP	GMP	XMP	AMP	UMP
**Solid**					
European oyster (*Ostrea edulis*)	30.4	17.6	n.d	94.0	123.7
	± 2.7	± 1.8	–	± 9.3	± 9.7
Pacific oyster (*Crassostrea gigas*)	15.1	9.2	n.d	42.4	89.4
	± 1.2	± 0.6	–	± 2.9	± 14.3
**Liquid**					
European oyster (*Ostrea edulis*)	6.9	n.d	n.d	n.d	9.8
	± 0.7	–	–	–	± 0.5
Pacific oyster (*Crassostrea gigas*)	n.d	n.d	n.d	n.d	n.d
	–	–	–	–	–

*n.d.* = not detected, i.e. < limit of detection (LoD) ≤ 1 μM.

## Discussion

Gauging from the twostep fermentation process behind genuine champagnes, as well as other non-authentic champagnes from other regions that use the traditional *méthode Champenoise*, with an extended yeast contact we would expect that champagnes hold a certain umami potential. It appears from our study that the champagnes do contain umami-tasting FAA and those with the longer yeast contact have the highest concentrations of both umami tasting Glu and overall total amount of FAA. However, in all cases the concentrations are found to be below the taste threshold of basal umami taste. In contrast, high levels of free glutamate were found in the two species of oysters that in addition contain high levels of inosinate and adenylate and are therefore cable of eliciting synergistic umami taste by themselves without champagne. Still, as we will discuss in the following, the umami taste compounds found in oysters will impact the perceived umami taste sensation when ingesting champagne with oysters, rendering them good companions in terms of umami synergy.

There is only little quantitative information available in the scientific literature regarding chemical analysis of champagnes and oysters with respect the compounds that elicit umami taste. It is, however, likely that both champagne companies and oyster producers have non-disclosed data for internal company use. Some sparse data are available regarding FAA in Pacific oysters^33–38^ but to the best of our knowledge no data has been reported for the content of free nucleotides. Similarly, there appears to be no previously published data for FAA and free nucleotides in the European oyster. Regarding champagnes, some published data are available of the FAA profile^39^ whereas there are more data for other types of fermented beverages such as wines and sake^40–42^. The occurrence of free nucleotides in champagne aged on lees for 8 years has been found to be rather low, being 0.005–0.01 mg/100 g for IMP; 0.01–0.05 mg/100 mL for GMP and AMP, and with XMP below detection threshold^43^. Previously reported contents of nucleotides in champagne wines are thereby lower than the taste threshold determined by Maga^32^. We shall therefore not be further concerned with free nucleotides in champagne and assume that the dominant contribution of nucleotides in the pairing of oysters and champagne is derived from the oysters.

### Contents of free umami amino acids and nucleotides

#### Champagnes

From the data in Table 1 it is found that the content of Glu across samples of champagne where Glu was identified, lies in the interval 1.4–7.5 mg/100 mL where the highest content was found in the older champagnes (vintage 2000–2008) aged on lees for 11–19 years. These findings are consistent with the work by Le Menn et al.^39^ who found Glu contents in a variety of champagnes aged on lees champagne in the interval of 1.7–5.6 mg/100 mL, also reporting some of the higher contents in the older champagnes. Other studies reporting the free amino acid content of wine found a content > 1 mg/100 mL^40^ and 4.7–16.8 mg/100 mL^44^. Hence, the highest concentrations found in the present study is in accordance with previous findings of other authors^39^ and can evidently be concluded to be below the umami taste threshold of 29–30 mg/100 mL^30, 32^. Champagne should therefore theoretically not elicit umami taste when consumed by itself, which may in fact explain the emergent deliciousness once paired with oysters. The pairing thus enables an umami taste well above the taste threshold due to the contribution from free Glu from both sources in combination with GMP and IMP from the oysters, rendering the synergistic umami effect as we will describe in detail below. In addition, the vast contribution of umami components from the oysters may also enhance sweet tasting FAA from the champagne (i.e., Ala), while masking the bitter ones (i.e., Arg).

The sum of FAA in the champagnes in the current study was found on average to be 65 mg/100 mL which is consistent with the content of 76.2 mg/100 mL reported by Le Menn et al.^39^. Correlation analysis in the same study by Le Menn between wine age and amino acid concentration did not yield strong correlations but did obtain the highest score for Asp (R^2^ = 0.39) being positively correlated to wine age. Although no clear correlation between wine age and sum of FAA was found, groupings of data showed that higher wine age correlates with wines with highest concentration of total FAA^39^.

#### Oysters

The present study provides for the first time a chemical analysis of the European oyster *Ostrea edulis*. The samples were collected in Limfjorden in the Northern part of Denmark, which is the largest remaining, natural terroir in the world for the European oysters. However, possibly due to climate changes, populations of *Crassostrea gigas* have since the mid-1990s massively invaded Limfjorden as well as other Nordic shallow water habitats^45^ and since 2017 established a self-sustaining population. There are growing concerns regarding the future of the indigenous European oyster whose habitats become overgrown by Pacific oysters. Attempts are currently made to cope with this problem by establishment of managerial practices for harvesting and possible uses of the invasive species as a new food source^46^. The results reported in the present paper may be useful for evaluating the taste qualities of *Crassostrea gigas* against *Ostrea edulis* in the context of future commercial and sustainable uses of local oyster populations.

From the data presented in Tables 2 and 3 it is seen that the analysed oysters contain fair amounts of both free Glu and free nucleotides. The contents of Glu were found to be 160 mg/100 g and 257 mg/100 g for *Crassostrea gigas* and *Ostrea edulis*, respectively. These findings are somewhat in consistence with Yuasa et al.^38^ who found a Glu content of 126 mg/100 g in *Crassostrea gigas* and 146 mg/100 g in *Crassostrea nippona.* Yoneda et al.^35^ also investigated the content of Glu in *Crassostrea gigas* and found it to be 40–190 mg/100 g, depending on season, being highest in the summer. The oysters investigated in the present study were found to be in the middle of this interval, which may account for the fact that they were harvested in late fall. A similar content was documented in *Crassostrea gigas* (whole body) as reported by Tanimoto et al.^36^ who found the content of Glu to be 181 mg/100 g, the value being dependent on storage period, decreasing during a period of up to 7 days. In soaking water after seven days at 3 °C, Glu was found to be 170 mg/100 g. Hosoi et al.^34^ reported a somewhat lower content of Glu in Pacific oyster, around 100 mg/100 g (13.3 μmol/g dry weight).

Apart from the time of year of harvest of the oysters, variance in the amount of FAA, and particular Glu, may also be due to the composition of the water they are harvested in. The source for FAA in fresh oysters is from the pool of FAA that oysters naturally have in their tissues and the water around the organs in the shell, and the same is the case for free nucleotides. Some of these compounds are derived from both the algae ingested by the oysters and the FAA in the ambient seawater. It is well known that high salinity increases the concentration of the osmolytes, most prominently FAA, in the tissues of the oysters, and that microalgae constitute a rich source of macro- and micronutrients for bivalves like oysters^47, 48^. In the oyster tissue the FAA and nucleotides, together with other small solutes, act as osmolytes to counterbalance the salinity of the ambient water. More of these solutes are hence expected in oysters from more salty waters. This physical–chemical effect along with the microalgae occurrence in the seawater are hence factors that determine the *merroir*^31^. In our study, this was accounted for by retrieving the two different species of oysters in the exact same location. The differences found in the two species may therefore be ascribed to the biological nature of the oysters itself and not their surroundings. The effect of the environment is used commercially when farming oysters that are grown in special *merroirs*^31^. An interesting example in the present context is a product called *Umami oyster* that is of *Crassostrea gigas* variety. It is derived from Ireland but farmed in the fjords of Zealand, a western province of The Netherlands. The producers supposedly transplant the oysters several times to different locations in the fjords in order to optimize flavour via exposing the oysters to different habitats with varying salinity and microorganisms. It is however not certain, that it is umami specific amino acids which exhibit osmolytic functions. Hosoi et al.^34^ investigated the effect of salinity changes on FAA content in Pacific oysters and found that Gly, Ala, Pro, and Arg experienced the greatest changes in content whereas Asp and Glu did not increase significantly, suggesting that they are not involved in the adaption to hyper-osmolality.

### Umami synergy in champagne-oyster companionship

In order to compare and evaluate the results for our measurements of FAA and nucleotides in the context of perceived umami taste it is useful to consider the thresholds for human taste perception for the umami compounds Glu, IMP, and GMP. Due to substantial variations among individuals as well as the way these thresholds are defined, different studies report rather different threshold values^32, 49–51^ The ISO standard for the lower detection value of Glu for qualifying for being member of a taste panel is set to 29 mg/100 g^30^ which is in consistence with what is reported in Maga^32^. The authoritative review by Maga^32^; see also^25, 52^), is of particular interest since it also gives threshold values for combinations of Glu with IMP and Glu with GMP. The actual taste intensity is often described by the non-linear empirical formula,

1 $$y = u + \, \gamma uv,$$

where *u* and *v* are the concentrations in mg/100 mL of Glu and IMP, respectively, in the aqueous mixture^25, 52^. γ = 1.218 is an empirically determined constant, and *y* is to be interpreted as the concentration of pure Glu in water which would effectively lead to the same intensity as the mixture of Glu and IMP in question. The synergy constants for the other free nucleotides can be estimated based on their relative taste intensity to IMP from Yamaguchi^24^ and are for GMP, γ = 2.801, for AMP, γ = 0.219, and for XMP, γ = 0.743 and their contributions can thus be included in Eq. (1). From this equation follows, that even if the concentration of Glu is considerably below the taste threshold, a very small amount of added IMP or GMP can bring the taste intensity of the mixture above the threshold.

Equation (1) can be generalized also to include Asp but since the umami potential of free Asp is much less (8%) than that of Glu^52^ we shall here only be concerned with Glu. According to Maga^32^, the taste threshold in water for a single component is for Glu 30 mg/100 g, for IMP 12 mg/100 g, for GMP 3.5 mg/100 g, and for equal amounts of Glu and IMP or Glu and GMP in a mixture the threshold is down to 0.1 mg/100 g and 0.03, respectively, illustrating the immense power of umami synergy.

The data presented in Tables 1, 2 and 3 show that when it comes to umami taste, pairing oysters with champagne provides the two necessary components, free glutamate (basal umami) and inosinate and guanylate (synergetic umami), in order to elicit an enhanced umami taste. It could be argued, that oysters alone elicit synergistic umami due to their large contents of both glutamate and nucleotides, and that champagne with its relatively modest contents of glutamate would not add much to this. However, the very fact that champagnes have values of free Glu below the taste threshold lends support to our hypothesis that champagne is a good companion to oysters. Since oysters and champagne are not ingested in the same mouthful it is likely that a sip of champagne subsequent to eating, masticating, and swallowing a raw oyster will, via its content of free glutamate, interact synergistically with traces of free inosinate in the saliva remaining from the ingested oyster, cf. Eq. (1). This leads to the well-known lingering of the umami taste^7^ along with mouthfullness and a deep savoury flavour and may be exemplified by a calculation example of the synergistic effect.

To calculate an estimate of the synergistic effect of umami-taste pairing, i.e., the value of *y* in Eq. (1), by combining a beverage with a certain content of free Glu with oysters or other foods, we have to make an assumption about a realistic volume mixing ratio in the oral cavity. We imagine the following situation: after having masticated and swallowed the oyster, a sip of champagne (about 20 mL) is ingested. We will assume that the volumes of oyster taste compounds left in aqueous saliva solution make up a volume fraction of 2.5–5% of this saliva when mixed with the ingested champagne. Based on these realistic assumptions we find, using Eq. (1), for a volume fraction of 5% that the champagnes as the ones in Table 1 with only free glutamate and no free nucleotides of their own need to have a minimum free Glu content of *champagne* (*u*_*min*._) = 0.2 mg/100 mL for *Crassostrea gigas* and *champagne* (*u*_*min*._) = a negative value/diluting effect from champagne for *Ostrea edulis* in order to bring the combination of champagne and oysters above the umami taste threshold of 30 mg/100 mL (equivalent concentration of free Glu). If a corresponding calculation is carried out for caviar, also considered to be a good companion with champagne, the champagne must be able to contain as much as 27.1 mg/100 mL, which for this case may be explained by the lack of nucleotides in caviar. Although synergistic umami effect may not bring the umami taste above the taste threshold for these specific combinations, other seafood may do, for instance scallops, which would allow champagnes with a content of above 3.6 mg/100 mL to facilitate perceived umami taste that applied for most of the champagnes (C01–C12, Table 1) included in this study, but not for the reference sample, the Verdi sparkling wine. As these calculations may point to the fact that synergistic umami effects may be in effect for combining oysters and champagne, it is however highly dependent on the ratio of the two contributions. Performing the same calculation as above for the two species of oysters but decreasing the estimated food contribution left in the mouth to only 2.5% will yield *champagne* (*u*_*min*._) = 9.1 mg/100 mL for *Crassostrea gigas* and *champagne* (*u*_*min*._) = 1.8 mg/100 mL for *Ostrea edulis*, rendering *Ostrea edulis* capable of inducing synergistic umami taste, but not *Crassostrea gigas,* in combination with the champagnes included in the present study. Thus, the actual ratio of champagne-oysters present inside the mouth, i.e., the degree to which food residues remain in the mouth, in addition to the quality (i.e. content of Glu) of both the champagne and the oyster will influence the perceived umami sensation. Ideally, this should be validated by a sensory panel conducting a sensory descriptive analysis.

### Other free amino acid taste contributions

**Figure 2:**
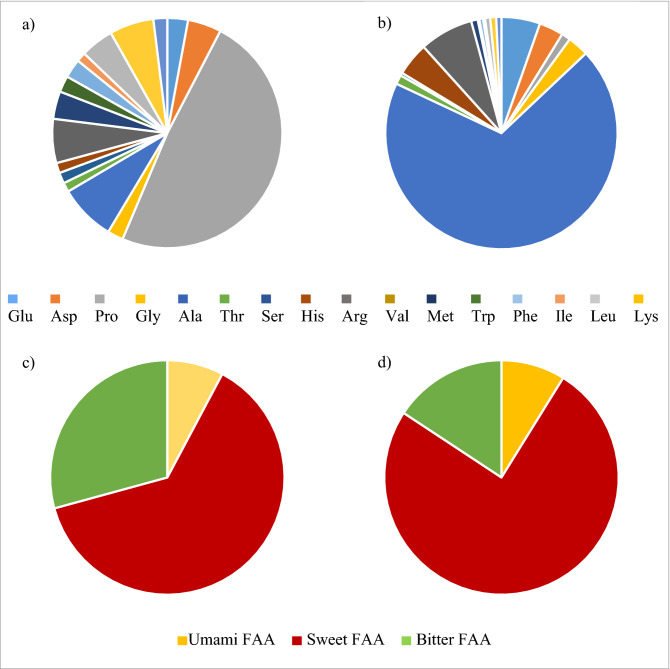
Amino acid profile of FAA (n = 16), FAA Tyr is not included as it is neutral in taste. FAA of oyster (*Ostrea edulis* and *Crassostrea gigas*) and average of champagnes. In the top: (**a**) and (**b**) display the distribution of specific amino acids based on an average for champagne and oysters, respectively. In the bottom: (**c**) and (**d**) display the distribution of amino acid taste property based on an average for champagne and oysters, respectively (yellow = umami taste, red = sweet taste, green = bitter taste).

In addition to umami taste compounds, sweet tasting amino acids were also quantified for oysters. A trait of oysters seems to be a high content of sweet tasting Ala which comprise roughly 3/4 of the total amount of FAA, whereas champagne in general has an abundance of sweet tasting amino acid Pro, in average 1/2 compared to the other amino acids (cf. Fig. 2a,b).

As seen from Fig. 2 it is evident that sweet tasting amino acids are a dominant part of the oyster taste profile. This may be a surprise, since it is not this taste attribute that first comes to mind when consuming oysters. This may however be explained by the fact, that some sweet tasting amino acids have umami tasting properties when present in high amounts. This has been found to be the case for Gly and Ala^53^ and Ser and Gln^54^.

### Other umami taste compounds

Despite its relatively low glutamate content, champagne might in fact possess a greater umami potential by itself than considered in the present paper. Other umami eliciting compounds than glutamate and nucleotides, as those presented in a recent review article by Wang et al.^55^, may play a role in the synergistic taste enhancement perceived when pairing oysters with champagne. For instance, glutathione compounds are examples of such compounds. Marchand and Revel^56^ investigated glutathione derivatives in must and wine and identified small amounts of γ-glutamylcystine (0.6–1.3 mg/L), while reduced glutathione was determined to be 1.8–6.6 mg/L in French dry white wines. According to some studies, glutathione does not act synergistically with glutamate, but does on the other hand do so with certain nucleotides^57^, suggesting yet another reason for the enhanced effect when pairing champagnes with oysters. The oysters will enhance the below-taste threshold values of both Glu and possible glutathione, even though they do act synergistically with each other. Other umami taste compounds may be disodium succinate^58^, that has been reported to exist in seafood, in particular scallops and clams, and to some degree also in oysters^59^. However, to which degree it will affect glutamate and ribonucleotides mixtures is not well established and would require extensive both analytical chemical studies as well as sensory analyses.

## Conclusion

In the present paper we have focused attention on a possible scientific mechanism behind champagne and oysters being good companions and we have substantiated our hypothesis by concrete and quantitative measurements of compounds that are known to elicit umami taste and engage in umami synergy. Furthermore, we have provided several calculation examples relating the quantitative data to taste threshold and hypothetic real meal situations.

It is obvious, however, that there are other factors beyond umami synergy, which can influence the judgement of good pairing of champagnes with oysters. Properties of the champagne like minerality, brininess, and prickling acidity have been pointed out to play a role^26^, in particular how these properties couple to mouthfeel^60^. Another effect could be that the acidity of the champagne may, despite the buffering capacity of saliva, act to lower the pH in the saliva shifting the equilibrium between glutamic acid and deprotonated glutamic acid towards glutamate, and this ion is a requirement for stronger binding of the umami substances to the umami receptor^20^.

Based on the calculations, the different requirements to the champagne for entering an effective umami synergy and how it depends on the choice of oysters are revealed. Pairing champagne with oysters containing a higher amount of umami compounds will induce umami synergy with champagnes being lower in Glu (i.e., rested for a shorter time on the lees). This was specifically illustrated by the difference between the two species of oysters, *Ostrea edulis* and *Crassostrea gigas,* where it was found that *Ostrea edulis* contains 50% more free inosinate and 60% more Glu than *Crassostrea gigas.* We wish to point out that this may be a reason why some tasters prefer the European oyster over the Pacific oyster. Nonetheless, it is obvious that champagne in terms of umami taste has more to gain from this pairing compared to the oysters.

## Materials and methods

### Samples

All champagnes and sparkling wine are commercially available products (see Table 1) and have been supplied by a range of individuals and companies. After sampling from the bottles in 15 ml vials the samples were kept at − 20 °C until analysis.

Both types of oysters, European oyster (*Ostrea edulis,* size AA) and Pacific oyster (*Crassostrea gigas*, mixed sizes) were harvested at the end of January 2019 by a fisherman skilled in the art in the same fishing area ground in Limfjorden, Denmark, Region 34 in Løgstør Bredning that is part of the Northeastern Atlantic (FAO27). The oysters were packaged at the fishing site and subsequently kept cold and transported in the usual commercially applied braided wooden boxes to be received and prepared in the laboratory within 2 days. Three out of six most similarly looking and non-damaged oysters of each species were used for analysis. After shucking, the water in the shells (liquid) and the drained tissue (solid) were collected separately for each type of oyster. The liquid part both consisted of the water in the shell after shucking and the water drained from the solid part 5 min after shucking. Liquid and solid material from the three oysters of each type were mixed and homogenized for each type of oyster, yielding a solid and a liquid sample from each type of oysters, each based on three specimens. The solid part was freeze dried and minced prior to homogenization. All samples were stored at − 40 °C until further analysis.

### Chemicals

Amino acid standard mix containing alanine (Ala), glycine (Gly), valine (Val), leucine (Leu), isoleucine (Ile), threonine (Thr), serine (Ser), proline (Pro), aspartic acid (Asp), methionine (Met), 4-hydroxyproline (Hyp), glutamic acid (Glu), phenylalanine (Phe), lysine (Lys), histidine (His), tyrosine (Tyr), tryptophan (Trp), and cystine (C–C) was purchased from Phenomenex, Torrance, USA. Solvents used were HPLC grade and purchased from Sigma-Aldrich, Copenhagen, Denmark.

5′-mononucleotide standards including adenosine 5′-monophosphate disodium salt (AMP, ≥ 99.0%), guanosine 5′- monophosphate disodium salt hydrate (GMP, ≥ 99%), and inosine 5′-monophosphate disodium salt hydrate (IMP, ≥ 99.0%) were obtained from Sigma-Aldrich, Copenhagen, Denmark. Xanthosine 5′-monophosphate disodium salt (XMP, ≥ 98%) and uridine 5′-monophosphate disodium salt (UMP, ≥ 99%) were obtained from Carbosynth, Compton, UK.

Ultrapure Milli-Q water (Milli-Q system, Millipore, Bedford, MA, USA) was used throughout the study.

### Sample preparation for free amino acids extraction

#### Champagne, wine and liquid oyster samples

The free amino acid extraction procedure of liquid samples followed Hildebrand et al.^61^ with some few modifications. The liquid sample volumes were mixed 1:1 with 12.5% trichloroacetic acid and kept in a refrigerator (5 °C) for 2 h. The samples were centrifuged at 15,000 × g for 20 min (Thermo Scientific Micro 17 CF Centrifuge) and the supernatant was separated and neutralized with 1 M sodium hydroxide. The neutralized samples were then diluted with Milli-Q water, added internal standard (aminocaproic acid) and filtered through 0.2 µm syringe filter (Tuberculin 1 mL syringes with 4 mm syringe filters, RC membrane, 0.2 µm, Phenomenex) into HPLC glass vials (Screw Cap Vials, clear, Agilent). The filtered samples were kept at freeze storage (− 20 °C) until analysis by UHPLC.

#### Solid oyster samples

The extraction procedure was applied according to optimized conditions described by Poojary et al.^62^ with some few modifications. Freeze-dried solid samples (5 g) were ground (Easy Grind, 100 W, OBH Nordica) at max speed for 1 min obtaining a homogenized powder. The homogenized samples (500 mg) were added to 25 mL of 25% acetonitrile added Milli-Q water. The sample solutions were stirred with a magnet bead at 585 rpm at room temperature (20 ± 0.5 °C) for 3 h. The sample extracts were then precipitated using the following technique: Samples were placed in a refrigerator at 5 °C for 20 min before centrifugation at 15,000 × g for 20 min (Thermo Fisher Scientific). After centrifugation, the supernatant was taken and added 100% acetonitrile and placed in a refrigerator at 5 °C for 20 min before the second centrifugation at 15,000 g for 20 min. Samples were then diluted with Milli-Q water and the samples were then diluted with Milli-Q water and added an internal standard, filtered, and stored until analysis as described above.

### Free amino acid analysis by UHPLC

The amino acid analysis was carried out according to the method by Hildebrand et al.^61^, using an ultra-high performance liquid chromatograph (UHPLC) (UltiMate 3000 UHPLC, Thermo Fischer Scientific, CA, USA) with a reversed-phase UHPLC column (Advanced Bio AAA column, Agilent, Glostrup, Denmark 3.0 × 100 mm, 2.7 μm particle size, with a guard cartridge) and fluorescence detection (FLD). Primary amino acids were derivatized with a solution of 7.5 mM o-phthaldialdehyde (OPA)/225 mM 3-mercapto-propionic acid (MPA) prepared in 0.1 M borate buffer. OPA/MPA solution was prepared freshly and used within 8 h. Secondary amino acid (Pro) were derivatized with 9.7 mM 9-fluorenyl-methyl chloroformate solution prepared in acetonitrile. A binary gradient system was applied with a mobile phase consisting of 10 mM Na_2_HPO_4_, 10 mM Na_2_B_4_O_7_, and 0.5 mM sodium azide (pH 8.2, mobile phase A); and acetonitrile mixed with methanol and Milli-Q water in ratio 45:45:10 v/v/v (mobile phase B). The mobile phase was filtered through a nylon membrane filter paper (0.22 μm, 47 mm, Phenomenex). The following instrumental settings were applied: Column oven temperature was set to 40 °C; flow rate of 0.62 ml/min using the following gradient conditions: 0 min 2% B, 0.35 min 2% B, 13.4 min 57% B, 13.5 min 100% B, 15.7 min 100% B, 15.8 min 2% B, and 18 min 2% B; data collection rate was set to 25 Hz and response time was 0.8 s. The FLD excitation and emission wavelengths was 340 nm and 450 nm at 0–10.4 min and 260 nm and 325 nm at 10.4–18 min. A standard curve (5–100 μM) was constructed using amino acid standard mix with added aminocaproic acid. All samples were analysed in triplicate. Amino acid concentrations were calculated as mg amino acid per 100 mL beverage.

### Sample preparation and 5′-mononucleotide analysis

The extraction procedure applied followed a previously described method in Schmidt et al.^63^. The freeze-dried samples were homogenized as described above. Samples (500 mg) were added into 15 mL screw cap flasks together with 10 mL boiling Milli-Q water. The sample solutions were agitated in a temperature-controlled (± 0.1 °C) boiling water bath for 1 min. The samples were subsequently sonicated in an ultrasound bath (Ultrasonic cleaner, Branson 2210E-MT, Branson Ultrasonics, Danbury, USA) for 15 min and put to rest overnight (20 h) in a refrigerator (5 °C). The next day the samples were centrifuged for 20 min at 12,000 × g (MicroCL 17 centrifuge, Thermo Fisher Scientific). The supernatant was retrieved and filtered through a 0.22-μm membrane filter into an HPLC glass vial and kept at − 20 °C until analysis.

### 5′-Mononucleotide analysis by HPLC

5′-Mononucleotide analysis was carried out using an Agilent 1200 HPLC (Agilent Technologies, CA, USA) equipped with a diode array detector. Nucleotides were separated using a SUPELCOSIL LC-18-T HPLC column (25 cm length × 4.6 mm internal diameter × 5 μm particle size, Sigma-Aldrich, Steinheim, Germany) at ambient temperature and detected at 257 nm. The method for separation of nucleotides was employed according to optimized results by Poojary et al.^62^ also applied in Schmidt et al.^63^. Mobile phase A consisted of a 50 mM KH_2_PO_4_ buffer where pH was adjusted with 10% KOH (w/v) to reach pH 4.8. Mobile phase B consisted of 100% methanol. Gradient conditions were as follows: 0–10 min 0% B, 28–45 min 20% B, and 46–61 min 0% B, and the flow rate was 0.5 mL/min. A standard curve (5–100 μM) was constructed using standards for quantification of nucleotides, and all sample analyses were performed in triplicates. 5′-Mononucleotide concentrations were calculated as mg of 5′-mononucleotide per 100 g of hydrated meat (mg/100 g) or per 100 mL liquid (mg/100 mL).

### Data analysis

The amino acid raw data was integrated in Chromeleon Chromatoraphy Data System Software (version 7.2.7, Thermo Fisher Scientific) program, and nucleotide data was processed in LC Systems software (Agilent). Both data outputs were further processed in Excel (version 2016) calculating sample concentrations. Multivariate Principal Component Analysis (PCA) was conducted in Latentix (version 2.12). Analysis of significance was performed in SAS JMP (SAS Institute, version 14.0.0) using Kruskal Wallis test appended with Steel’s control test for output measures displaying non-normal distribution, whereas 1-way analysis of variance appended with Dunn’s control test were applied for output measures displaying Gaussian distribution, using the sample of sparkling wine as control for beverage samples. Responses were evaluated by p-values, and a p-value < 0.05 was considered significant.
